# Supplementary material for: Transcriptome Landscape Variation in the Genus Thymus
Source: Genes (Basel). 2019 Aug 16;10(8):620. doi: 10.3390/genes10080620 (PMC6723042; doi:10.3390/genes10080620)
Supplement: Supplementary file 1 [file genes-10-00620-s001.pdf]

Supplementary Materials:

Table S1. Assembly statistics results of *Thymus* species.

Tools	Kmer Size	Species	Number of contigs	Total size (Mp)	Minimum length (bp)	Maximum length (bp)	Average length (bp)	L50 (bp)
Trinity	25	*T. daenensis*	91127	55.83	224	12126	614.35	819
		T. vulgaris	117835	68.35	224	14141	580.1	732
		T. lancifolius	123698	73.24	224	25032	592.09	767
		T. persicus	85084	58.47	224	13000	687.26	999
		T. pubescens	96976	59.99	224	15305	618.64	818
Bridger	25	*T. daenensis*	70626	56.14	201	14373	794.96	1350
		T. vulgaris	82450	68.78	201	17174	834.24	1414
		T. lancifolius	89526	72.02	201	26796	804.52	1416
		T. persicus	67166	59.58	201	21482	887.15	1500
		T. pubescens	67288	56.31	201	11592	836.99	1377
	27	*T. daenensis*	70235	54.72	201	13733	779.13	1316
		T. vulgaris	81814	67	201	15398	819.01	1396
		T. lancifolius	89516	70.67	201	20523	789.51	1393
		T. persicus	66716	58.04	201	20248	869.98	1474
		T. pubescens	66565	54.54	201	14125	819.48	1345
BinPacker	25	*T. daenensis*	66591	68.45	200	14373	1028.03	1553
		T. vulgaris	88953	94.52	200	17174	1062.68	1603
		T. lancifolius	94939	98.79	200	25544	1040.63	1634
		T. persicus	67179	75.24	200	21482	1120.01	1665
		T. pubescens	67809	73.72	200	11592	1087.3	1574

Table S2. Velvet/Oases assembly statistics results of *Thymus* species.

Species/K-mers		21	23	25	27	29	31	33	35	37	39	41	43	45	47	49	51	53	55	57	59	61
T. daenensis	Number of contigs	64407	63611	61900	60130	56679	54699	53888	53499	52686	52284	51817	51422	51103	50413	50156	50024	49953	49739	49921	49370	48985
	Total size (Mp)	56.31	56.61	55.16	54.64	51.09	48.69	47.78	47.29	45.93	45.6	44.67	43.83	43.06	42.43	41.6	41.42	41.24	41.13	40.4	39.48	38.43
	average lenght (bp)	874.36	890.06	891.18	908.85	901.47	890.32	886.68	884	871.8	872.3	862.11	852.39	842.64	841.81	829.46	828.05	825.73	827.11	809.42	799.68	784.71
	N50 lenght (bp)	1327	1343	1358	1384	1383	1375	1369	1364	1345	1341	1326	1310	1296	1305	1284	1285	1284	1295	1268	1257	1230
T. vulgaris	Number of contigs	88534	86637	84537	80270	74483	71518	70248	69325	68744	68430	67662	66852	66980	66396	66031	65765	65972	65585	65698	64711	64646
	Total size (Mp)	73.57	73.65	71.69	70.53	66.04	62.09	61.12	60.42	59.59	59.48	58.1	57.85	57.59	56.74	55.77	55.65	55.8	55.49	55.35	54.08	52.68
	average lenght (bp)	831.04	852.79	848.05	878.72	886.71	868.26	870.08	871.59	866.91	869.31	858.76	858.76	859.87	854.59	844.68	846.2	845.88	846.13	842.61	835.75	814.93
	N50 lenght (bp)	1259	1289	1286	1338	1364	1333	1345	1346	1347	1348	1334	1334	1339	1331	1323	1332	1342	1362	1353	1352	1323
T. lancifolius	Number of contigs	99808	98848	95024	91049	83022	80859	79882	78650	78013	77393	76437	76296	75789	74930	74324	74274	74090	74063	74088	73737	73396

	Total size (Mp)	84.08	85.41	84.11	81.83	74.21	71.58	70.67	69.39	69.04	68.43	67.61	67.22	67	65.82	64.83	64.51	64.6	64.47	63.88	62.72	61.4
	average lenght (bp)	842.5	864.1	885.17	898.8	893.91	885.26	884.73	882.35	885.08	884.21	884.54	881.14	884.08	878.49	872.38	868.61	871.99	870.59	862.3	850.64	836.63
	N50 lenght (bp)	1303	1334	1369	1405	1420	1416	1416	1410	1415	1420	1420	1413	1427	1412	1409	1411	1421	1429	1425	1414	1394
	Number of contigs	69437	64453	65231	61548	57545	57149	56241	55495	55238	55154	55206	54830	54804	54662	54427	54512	54655	54986	54966	54471	54769
T. persicus	Total size (Mp)	61.25	62.21	64.16	60.5	56.49	56.36	55.43	54.48	53.42	53.13	52.55	52.38	51.86	51.02	50.7	50.58	50.75	50.5	50.19	49.09	48.11
	average lenght (bp)	882.15	965.3	983.72	983.03	981.78	986.33	985.72	981.86	967.25	963.46	952.04	955.43	946.38	933.45	931.57	928.05	928.69	919.98	913.14	901.23	878.49
	N50 lenght (bp)	1357	1357	1498	1504	1502	1508	1502	1503	1479	1478	1457	1476	1460	1447	1443	1440	1449	1434	1426	1401	1375
	Number of contigs	61109	58280	55685	54687	51719	50534	49772	49324	48909	48511	48245	48042	48199	47688	47783	47850	48078	48105	48491	48306	47938
T. pubescens	Total size (Mp)	53.96	52.5	50.44	49.72	47.61	45.99	45.06	44.35	43.93	43.17	42.81	42.21	41.61	41.19	40.78	40.8	40.89	40.63	40.16	39.71	38.71
	average lenght (bp)	883.03	900.82	905.86	909.58	920.61	910.12	905.52	899.28	898.3	890.02	887.44	878.68	863.43	863.82	853.59	852.75	850.69	844.61	828.27	822.05	807
	N50	130	133	135	135	138	137	136	135	135	134	134	133	130	130	129	129	130	129	127	126	125

	lengh t (bp)	8	2	3	7	6	6	4	7	7	1	0	0	4	9	2	3	4	8	6	3	5

Table S3. The number of full length transcripts for three assemblers and five species.

Percent length coverage		100	90	80	70	60	50
	Trinity	3082	1114	883	866	905	1009
T. daenensis	Bridger	3374	1146	825	769	794	851
	BinPacker	3405	1156	842	777	791	826
	Trinity	3234	1217	1034	976	1005	1114
T. vulgaris	Bridger	3730	1258	901	812	841	840
	BinPacker	3806	1276	926	825	847	852
	Trinity	3269	1212	1044	963	993	1096
T. lancifolius	Bridger	3662	1239	910	819	866	861
	BinPacker	3722	1264	934	839	881	872
	Trinity	3549	1211	928	817	837	873
T. persicus	Bridger	3778	1204	872	729	728	712
	BinPacker	3815	1212	905	731	738	694
	Trinity	3445	1257	968	852	943	962
T. pubescens	Bridger	3544	1199	864	720	779	802
	BinPacker	3603	1198	877	752	791	807

Table S4. The contigs number of eleven genes involved in thymol and other monoterpene biosynthesis identified in *Thymus* transcriptomes.

TF families	*T. daenensis*	*T. vulgaris*	*T. lancifolius*	*T. persicus*	*T. pubescens*
**bHLH**	4630	6189	6533	4479	4631
**NAC**	2241	3234	3015	2316	2458
**MYB_related**	2232	3198	3099	2587	2649
**ARF**	2092	2018	2416	1796	1661
**WRKY**	2090	3011	2582	2246	2666
**ERF**	1672	2367	2313	1812	2108
**C2H2**	1583	2269	2449	1732	1768
**C3H**	1351	1855	1708	1458	1516
**FAR1**	1264	1752	1644	1315	1346
**B3**	1257	1773	1769	1494	1372
**HD-ZIP**	400	520	499	410	412
**G2-like**	78	86	103	99	76
**GRAS**	78	86	103	99	76
**GATA**	70	77	92	88	68
**TALE**	56	62	74	70	54
**LBD**	48	53	63	59	46
**TCP**	44	48	58	54	42
**SBP**	36	40	48	44	34
**AP2**	35	39	46	42	33
**CO-like**	31	34	41	37	29
**HB-other**	30	33	40	36	28
**NF-YB**	30	33	40	36	28
**MIKC_MADS**	26	29	34	30	24
**Nin-like**	26	29	34	30	24
**YABBY**	26	29	34	30	24

**Dof**	24	26	32	28	22
**HSF**	24	26	32	28	22
**NF-YC**	23	25	30	26	21
**CPP**	22	24	29	25	20
**E2F/DP**	20	22	26	22	18
**SRS**	20	22	26	22	18
**WOX**	20	22	26	22	18
**ARR-B**	19	21	25	21	17
**GRF**	19	21	25	21	17
**BES1**	17	19	22	18	15
**BBR-BPC**	16	18	21	17	14
**CAMTA**	15	17	20	16	13
**NF-YA**	14	15	18	14	12
**GeBP**	11	12	15	11	9
**LSD**	11	12	15	11	9
**STAT**	11	12	15	11	9
**DBB**	9	10	12	8	7
**M-type_MADS**	8	9	11	7	6
**NF-X1**	6	7	8	4	4
**ZF-HD**	5	6	7	3	3
**RAV**	4	4	5	1	2
**S1Fa-like**	4	4	5	1	2
**VOZ**	4	4	5	1	2
**Whirly**	4	4	5	1	2
**EIL**	3	3	4	0	1
**HRT-like**	3	3	4	0	1
**HB-PHD**	1	1	1	-	-
**SAP**	1	1	1	-	-

Table S5. The contigs number of eleven genes involved in thymol and other monoterpene biosynthesis identified in *Thymus* transcriptomes

Genes name	*T. daenensis*	*T. vulgaris*	*T. lancifolius*	*T. persicus*	*T. pubescence*
**DXR**	9	4	8	6	8
**DXS**	12	14	16	9	15
**MCT**	2	1	4	1	1
**CMK**	3	2	1	4	2
**MDS**	1	2	1	2	4
**HDS**	9	9	12	15	18
**HDR**	20	22	9	17	20
**IDI**	3	3	5	10	6
**GGPS**	6	7	4	9	9
**GDS**	1	2	2	3	2
**TPS**	15	25	9	21	9
**Total**	**81**	**91**	**80**	**97**	**94**

Table S6. Lengths of genes involved in thymol and other monoterpene biosynthesis identified in *Thymus* transcriptomes

**Genes name**	***T. daenensis***	***T. vulgaris***	***T. lancifolius***	***T. persicus***	***T. pubescence***	***Salvia miltiorrhiza***	***T. vulgaris***	***T. caespititius***
**DXR**	1422	1422	1422	1422	1422	1425	-	-
**DXS**	2122	2122	2122	2122	2122	2145	-	-
**MCT**	915	915	910	915	915	915	-	-
**CMK**	1188	1188	1188	1188	1188	1191	-	-
**MDS**	705	705	705	705	705	705	-	-
**HDS**	2229	2229	2229	2229	2229	2229	-	-
**HDR**	1389	1389	1389	1389	1389	1392	-	-
**IDI**	862	906	731	862	862	918	-	-
**GDS**	1275	1275	1167	816	1275	1275	-	-
**GGPS**	1085	1085	1085	1085	1085	1095	-	-
**TPS2**	1791	1615	1231	1733	1197	-	1791	
**TPS3**	1154	1788	1788	1788	1618	-	1788	
**TPS4**	1654	1665	1639	1060	1665	-	-	1665
**TPS5**	1747	1747	1747	1747	1747	-	1747	-

Table S7. The unigenes subjected to positive selection

T. daenensis	***T. lancifolius***	***T. persicus***	***T. pubescens***
Histidine kinase 5	ABC transporter G family member 6	ABC transporter I family member 1	Probable anion transporter 4
AT-hook motif nuclear-localized protein 8	AT-hook motif nuclear-localized protein S10	E3 ubiquitin-protein ligase ATL31	L-ascorbate peroxidase
Probable dolichyl pyrophosphate Glc1Man9GlcNAc2 alpha-1,3-glucosyltransferase	Probable anion transporter 4	Berberine bridge enzyme-like 21	Actin-related protein 3
Probable RNA-binding protein ARP1	Anaphase-promoting complex subunit 1	Transcription factor bHLH106	RING-H2 finger protein ATL38
Histone-lysine N-methyltransferase ATXR6	Transcription factor bHLH110	Chaperone protein ClpB	E3 ubiquitin-protein ligase BRE1-like 2
Probable BOI-related E3 ubiquitin-protein ligase 3	Transcription factor BPE, (bHLH 31)	Cyclic nucleotide-gated ion channel 2	Cytochrome P450 71A8
Chaperone protein ClpB1	Serine/threonine-protein kinase BRI1-like 2	Furcatin hydrolase	Receptor protein-tyrosine kinase CEPR1
CLP protease regulatory subunit CLPX2	Histone-lysine N-methyltransferase CLF	LanC-like protein GCL2	Cyclic nucleotide-gated ion channel 2
Protein CLT1, chloroplastic	CLP protease regulatory subunit CLPX2	Lysophospholipid acyltransferase LPEAT2	Protein CONTINUOUS VASCULAR RING 1
Glucan endo-1,3-beta-glucosidase 5	Protein CLT1, chloroplastic	Probable mediator of RNA polymerase II transcription subunit 26b	Glucan endo-1,3-beta-glucosidase 6
F-box protein	Ubiquinone biosynthesis O-methyltransferase	Pentatricopeptide repeat-containing protein	F-box protein

Gibberellin 20 oxidase 2	Putative protease Do-like 14	Serine/threonine-protein phosphatase 7	Heat shock 70 kDa protein 8
ARF guanine-nucleotide exchange factor GNOM	Zinc finger BED domain-containing protein DAYSLEEPER	Protein NRT1/ PTR FAMILY 7.3	Probable serine/threonine protein kinase IRE
Copper-transporting ATPase HMA4	Elongator complex protein 1	Ribonuclease 3-like protein 2	Ent-kaurenoic acid oxidase 2
Kinesin-like protein KIN-12B	Embryogenesis-associated protein EMB8	NAD-dependent protein deacetylase SRT1	3-ketoacyl-CoA synthase 2
Transcription factor KUA1 (Myb-related protein H)	1-phosphatidylinositol-3-phosphate 5-kinase FAB1B	F-box protein SKIP23	Protein LAZ1 homolog 2
Transcription factor LHW (BHLH transcription factor delta)	F-box protein	Tubulin-folding cofactor D	L-type lectin-domain containing receptor kinase VII.1
Midasin (AtMDN1)	L-gulonolactone oxidase 3	Mitochondrial import inner membrane translocase subunit TIM8	RNA cytidine acetyltransferase 1
NADH dehydrogenase 1 alpha subcomplex subunit 13-B	Nuclear pore complex protein GP210	Probable LRR receptor-like serine/threonine-protein kinase	NADH-ubiquinone oxidoreductase chain 6
Protein phosphatase 2C 29	Type II inositol polyphosphate 5-phosphatase 15	Probable inactive receptor kinase	O-fucosyltransferase 2
GPI mannosyltransferase 1	Probable LRR receptor-like serine/threonine-protein kinase IRK	Protein YLS3 (Protein YELLOW-LEAF-SPECIFIC GENE 3)	OTU domain-containing protein
Pentatricopeptide repeat-containing protein	G-type lectin S-receptor-like serine/threonine-protein kinase LECRK1		Pectinesterase inhibitor 10
Respiratory burst oxidase homolog protein C	Probable 1-acyl-sn-glycerol-3-phosphate acyltransferase 4		Putative pentatricopeptide repeat-containing protein
G-type lectin S-receptor-like serine/threonine-protein kinase RLK1	MADS-box transcription factor 6		Pentatricopeptide repeat-containing protein
Receptor-like protein kinase 5	Mediator of RNA polymerase II transcription subunit 16		Pentatricopeptide repeat-containing protein
Disease resistance RPP8-like protein 3	Plastid division protein PDV1		Glucosidase 2 subunit beta (Glucosidase II subunit beta)
Subtilisin-like protease SBT5.4	Pentatricopeptide repeat-containing protein		Putative late blight resistance protein homolog R1A-6
Ethylene-responsive transcription factor SHINE 2	PRA1 family protein B4 (AtPRA1.B4)		DNA repair protein recA homolog 3
Single-stranded DNA-binding protein	Receptor-like cytosolic serine/threonine-protein kinase RBK2		Remorin 1.4
Thioredoxin-like 3-3 (Thioredoxin-like 1)	SCARECROW-LIKE protein 7		Probable disease resistance protein RXW24L

Beta-D-glucosyl crocetin beta-1,6-glucosyltransferase	Potassium channel SKOR (Stelar K(+) outward rectifying channel)		Tryptophan--tRNA ligase
Extra-large guanine nucleotide-binding protein 1	SEC1 family transport protein SLY1		Thylakoid lumenal protein TL20.3,
WEB family protein	Protein trichome birefringence-like 16		Ubiquitin-like domain-containing CTD phosphatase
Probable receptor-like serine/threonine-protein kinase	Ubiquitin carboxyl-terminal hydrolase 3		Leucine-rich repeat receptor-like serine/threonine-protein kinase
	Vesicle-associated membrane protein 711		PHD finger protein
	Vacuolar-processing enzyme (VPE)		
	Probable inactive receptor kinase		

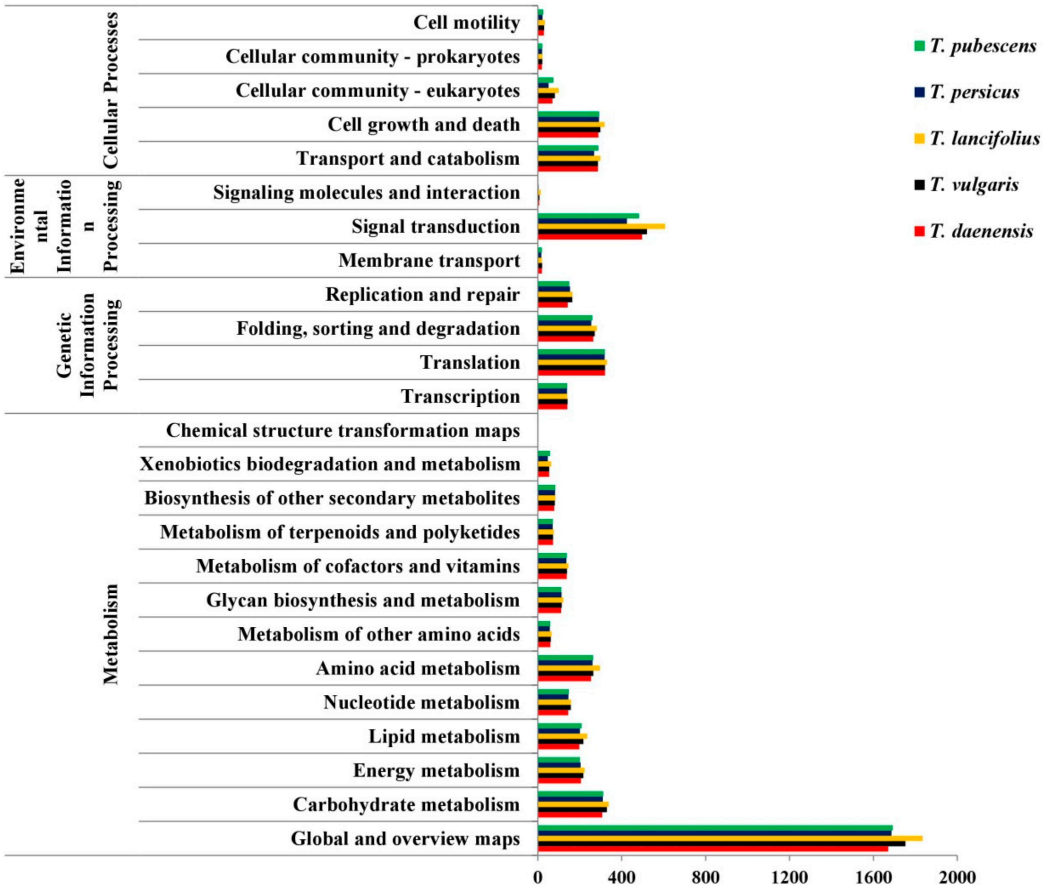

Figure S1. KEGG classification of unigenes for *Thymus* transcriptomes.

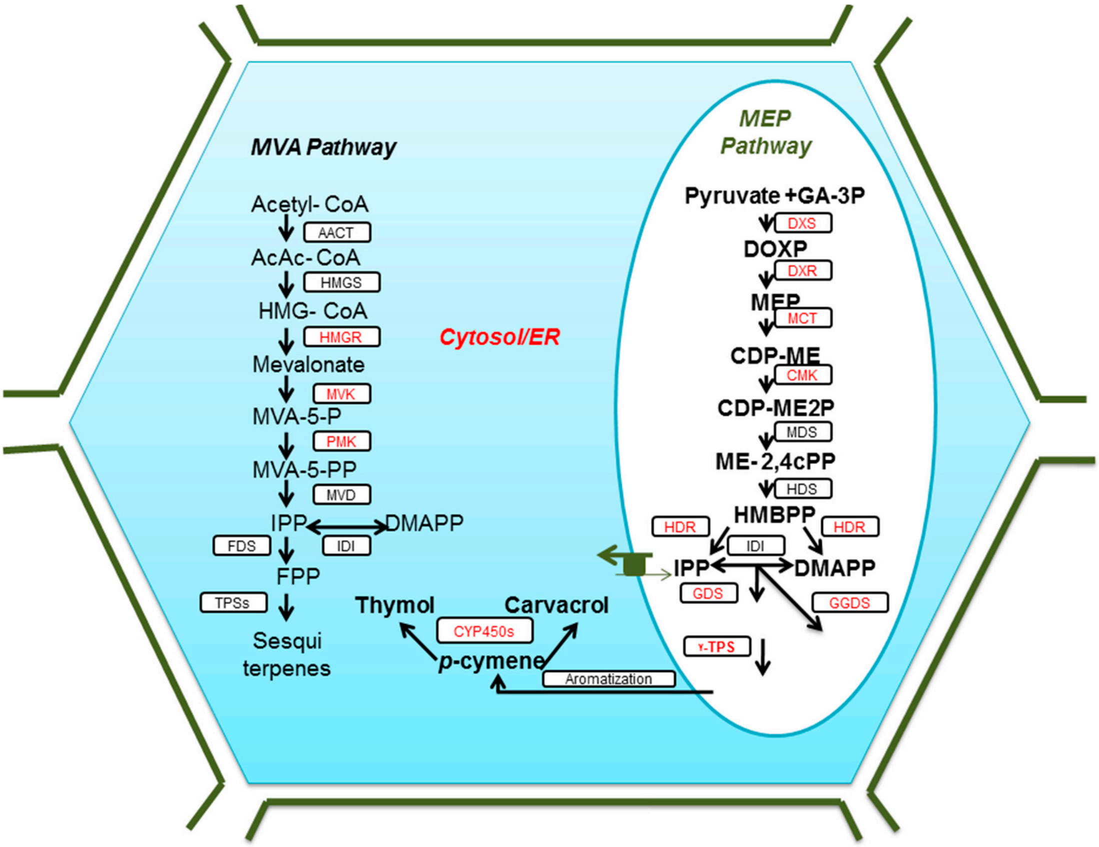

Figure S2. Schematic representation of thymol and carvacrol biosynthetic pathway.

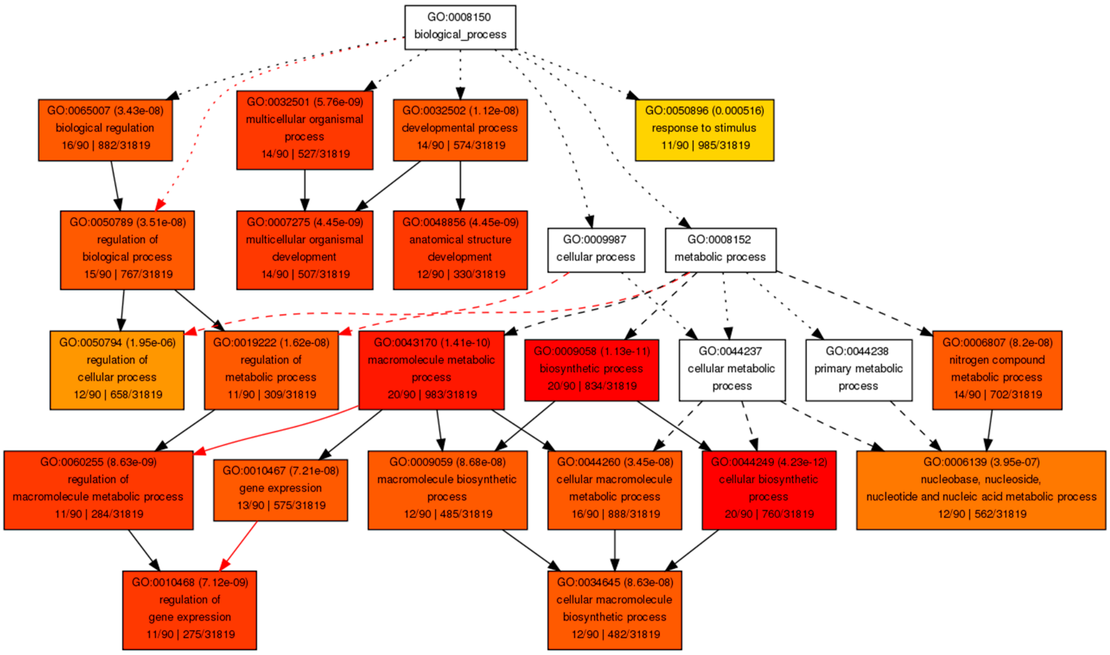

Figure S3. GO enrichment of PSGs related to ecological adaptation in *Thymus* species.

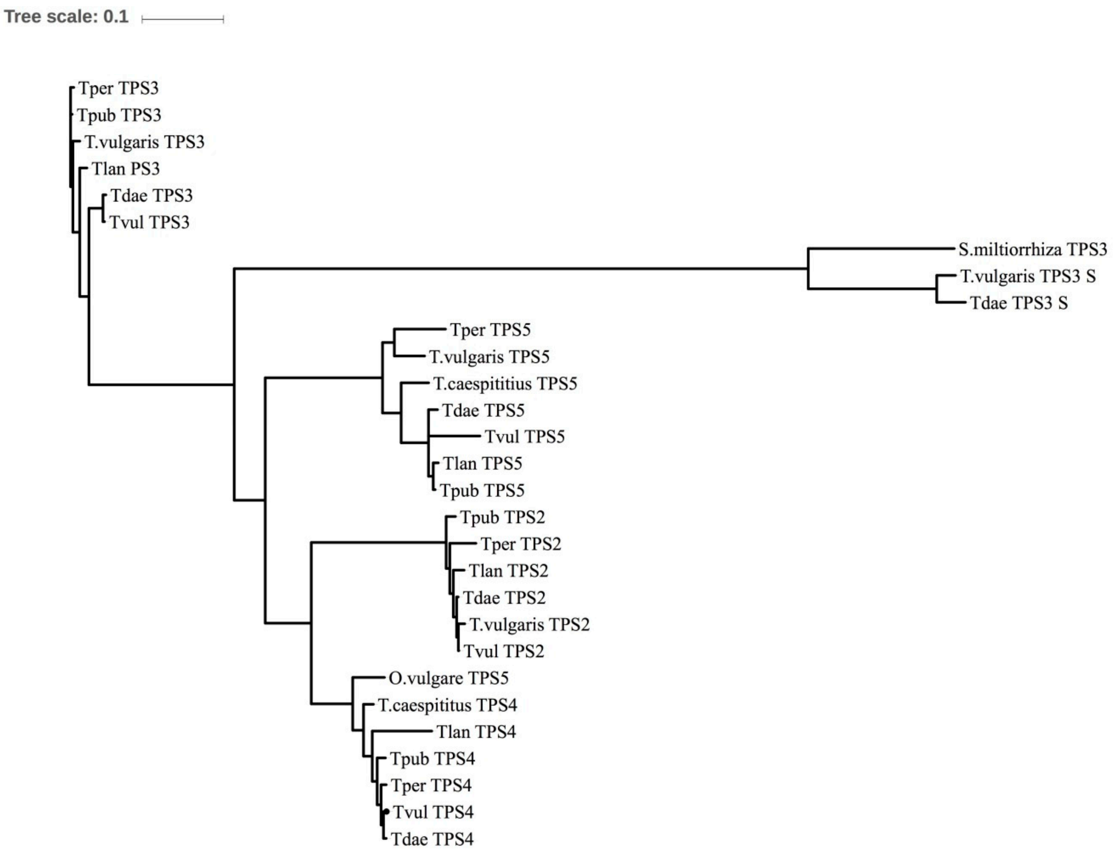

Figure S4. Maximum likelihood phylogeny of putative TPS genes in *Thymus* species.
